# Annexin A2 at the Interface of Actin and Membrane Dynamics: A Focus on Its Roles in Endocytosis and Cell Polarization

**DOI:** 10.1155/2012/852430

**Published:** 2012-02-22

**Authors:** Adam G. Grieve, Stephen E. Moss, Matthew J. Hayes

**Affiliations:** ^1^Hubrecht Institute for Developmental Biology and Stem Cell Research, Uppsalalaan 8, 3584CT Utrecht, The Netherlands; ^2^Division of Cell Biology, UCL Institute of Ophthalmology, 11-43 Bath Street, EC1V 9EL London, UK

## Abstract

Annexins are a family of calcium- and phospholipid-binding proteins found in nearly all eukaryotes. They are structurally highly conserved and have been implicated in a wide range of cellular activities. In this paper, we focus on Annexin A2 (AnxA2). Altered expression of this protein has been identified in a wide variety of cancers, has also been found on the HIV particle, and has been implicated in the maturation of the virus. Recently, it has also been shown to have an important role in the establishment of normal apical polarity in epithelial cells. We synthesize here the known biochemical properties of this protein and the extensive literature concerning its involvement in the endocytic pathway. We stress the importance of AnxA2 as a platform for actin remodeling in the vicinity of dynamic cellular membranes, in the hope that this may shed light on the normal functions of the protein and its contribution to disease.

## 1. The Annexins

**Figure 1:**
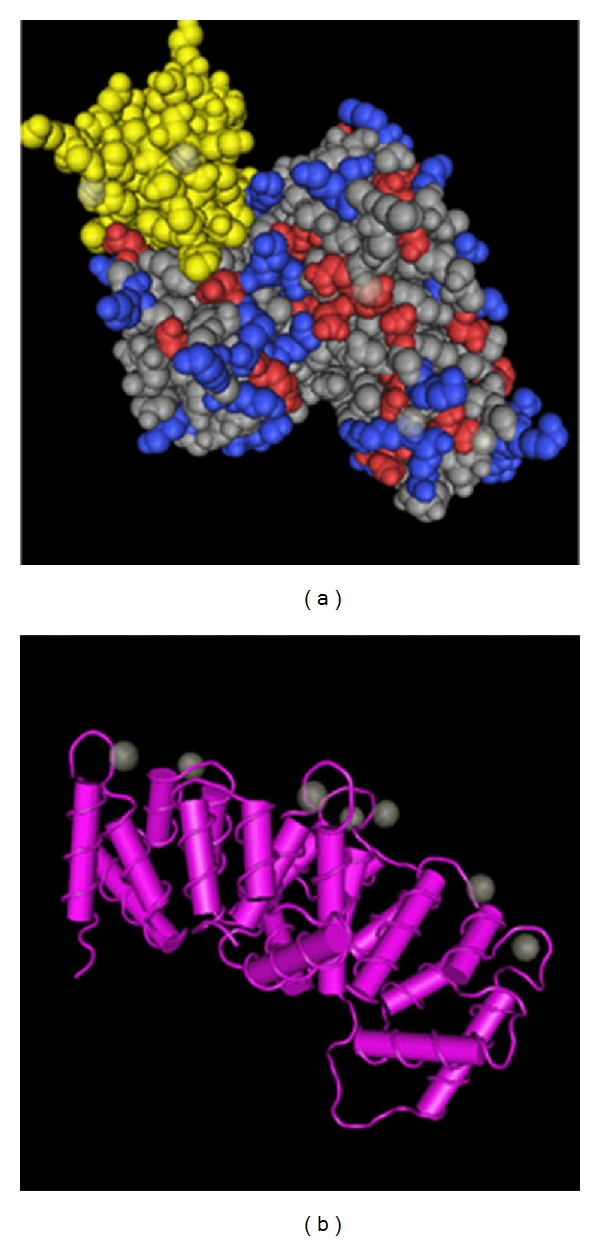
Structure of AnxA2. (a) Space filling view of AnxA2 (colours indicate hydrophobicity) looking “down” onto the membrane-binding surface of the molecule. One endonexin fold, repeated four times in AnxA2, is highlighted in yellow. (b) “Cartoon” of AnxA2 showing the molecule from the side. The protein is almost entirely made up of alpha-helices. The position of coordinated calcium atoms is shown in green.

A number of reviews have already been written on this large family of proteins. For the sake of brevity, we can only cursorily describe these roles. The annexins are found in all multicellular and some single-cell eukaryotes. They are a highly conserved family of calcium and phospholipid-binding proteins usually comprising four repeats of the characteristic alpha-helical-rich endonexin fold and an N-terminal domain that is unique to each annexin [1–5], Figure 1. Individual annexins tend to show restricted expression in particular cell types, the so-called “annexin fingerprint”, and where they are expressed; they tend to be highly abundant.

In different phyla, they have evolved to perform a variety of cellular functions but all retain a set of core properties [2–7]. They bind dibasic metal ions (usually calcium) and associate with charged phospholipids. They sometimes form oligomers, which can associate into high-order pseudocrystalline arrays on the surface of membranes, somewhat reminiscent of viral capsids [1]. In other cases, they have been suggested to insert into membranes, potentially forming pores or indeed to pass through the membrane entirely to act as an extracellular protein, with functions that may have little to do with their intracellular roles. Membrane association is dependent upon lipid content, calcium concentration, pH, and secondary modifications such as phosphorylation/myristoylation and their associations with a wide selection of binding partners [1, 2].

 In the most general terms, they have been implicated in calcium sensing and homeostasis [8–10], pH sensing [11], actin binding and regulation [12, 13], cell signaling [14–16], and fibrin homeostasis [17–20]. This list is far from being complete and individual annexins may have overlapping or shared functions.

 Changes in their expression levels have been implicated in a number of human diseases, loosely termed “annexinopathies” (reviewed in [21–24]). In most cases, the role of the annexin in pathogenesis has been linked to a well-characterized extracellular function of the protein, for instance, as a viral receptor [25], in regulation of fibrin [17–20] or in autoimmune disease [25]. However, intracellular functions of the proteins are likely to be at least as important, for instance in their role in mineralization of bone and cartilage for example [26, 27] or in tumour development [28].

 In this paper, we shall concentrate on Annexin A2 (AnxA2), which, whilst being an archetypal annexin, has a number of distinct properties rendering it particularly apposite to regulate actin-associated cellular processes at dynamic membranes.

Importantly, of all the annexins, altered expression of AnxA2 is most correlated with disease progression. The protein has been found on the HIV particle and has been implicated in the maturation of the virus [29, 30]. Furthermore, it is been linked to carcinogenesis and the progression of invasive cancer [23, 24, 28], suggesting an AnxA2-specific function that is necessary for tumour development. Indeed, AnxA2 was first identified as a major substrate of the tyrosine kinase v-Src, the transforming gene product of the Rous sarcoma virus [31–34].

### 1.1. Membrane Recruitment of AnxA2

AnxA2 differs from other annexins in that its association to membranes is most sensitive to increasing calcium levels (only requiring 0.05 *μ*M calcium to drive membrane association) [9] and structurally, in that it has a unique and relatively short 36 aminoacid N-terminal domain. AnxA2 is mostly found in a stable heterotetramer with S100A10, a small, EF-hand protein that is unique within its own family in being able to bind calcium. This complex, consisting of two S100A10 and two AnxA2 molecules [34–36], has a higher affinity for calcium and phospholipid than the AnxA2 monomer alone [34] and is found associated with the cytoplasmic leaflet of the plasma membrane and specific membrane-bound structures, such as endosomes and transport intermediates [36, 37]. Ionophores and extracellular ligands which cause influx of calcium further recruit the AnxA2-S100A10 complex to these membrane sites, although it often remains associated with these membrane domains after the initial calcium pulse has subsided (our unpublished observations). This suggests that the initial calcium-dependent recruitment is followed by stabilization; either by protein-protein or protein-lipid interactions. This most likely includes association with the submembranous actin cytoskeleton known to be present on endosomes and the plasma membrane [38, 39].

AnxA2 has been shown to favour cholesterol-rich subdomains of membranes [40], suggesting a predilection for regions of the membrane with reduced fluidity. Furthermore, *in vitro*, AnxA2 seems to have affinity for most anionic phospholipids and displays a calcium-enhanced affinity for phosphatidylinositol 4,5-bisphosphate (PI(4,5)P2) [41–44], a lipid enriched at the plasma membrane and vesicles derived from it. This lipid preference is in line with its cellular location (exclusion from membranes of the endoplasmic reticulum, Golgi, mitochondria) and in association with the plasma membrane (in particular to the apical membrane in polarized cells) and specific membrane-bound compartments.

Accordingly, in cells lacking the inositol polyphosphate 5′-phosphatase OCRL1 (which has a preference for PI(4,5)P2, but also acts upon PI(3,4,5)P3), AnxA2 is recruited to vesicular structures that contain presumed PI(4,5)P2 accumulations [42, 45, 46]. These structures, so-called “rockets”, are highly motile; being driven around the cell by a rapidly growing, arborescent actin tail (sometimes called an actin comet) and are richly decorated with AnxA2. Depletion of AnxA2 or inhibition of PI(4,5)P2 production in cells lacking OCRL1 blocks the formation of these rockets.

This observation demonstrates the properties we believe AnxA2 is critical for, that is, the remodeling of actin driving membrane rearrangements.

### 1.2. Annexin A2 and Actin

The ability of the AnxA2 and the AnxA2-S100A10 tetrameric complex to bind and bundle filamentous actin (F-actin) was first identified using proteins purified from the brush-borders of porcine epithelial cells [47] and from A431 cells [48]. The affinity of AnxA2 for actin and its requirement for calcium was further refined using protein purified from bovine lung [49] and a putative actin-binding nona-peptide was identified in the C-terminus of the protein [50]. The tetrameric complex is able to bundle F-actin at near physiological calcium concentrations (0.5 *μ*M calcium) and gives rise to a 1 : 1.9 stoichiometry of annexin bound to actin. Interestingly, the bundling activity of the tetrameric complex was found to be decreased by phosphorylation of the protein on Tyr23, the site favoured by the tyrosine kinase Src, suggesting a possible role for Src in the association of AnxA2 with actin [51].

At micromolar calcium concentrations *in vitro*, the AnxA2 monomer alone (without associated S100A10) is able to “cap” the fast-growing “barbed” end of the growing actin filament and possibly also interacts directly with G-actin monomers. Thus, AnxA2 monomer not only has the capacity to bundle preformed filaments, but may regulate the growth of newly forming ones [52].

Whilst the protein may perform this bundling and bridging function we suspect it is not its primary function. Accordingly, AnxA2 is not usually seen “coating” large actin bundles, such as stress-fibres and filopodia, as would be expected for a conventional actin bundling protein. Instead, AnxA2 is likely to have a more precise role in regulating actin cytoskeletal rearrangements. Consistently, *in vivo*, AnxA2 localises to regions of free-barbed ends (identified with labeled monomeric actin units) [52].

**Figure 2:**
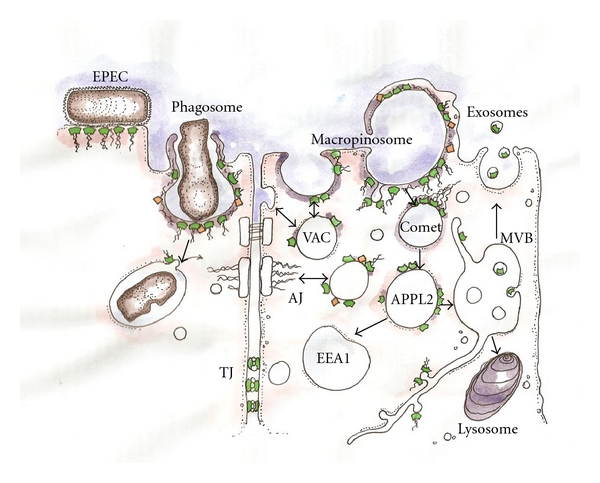
Recruitment of AnxA2 to several sites in the endocytic pathway. AnxA2 is shown as little green structures, filamentous actin as wavy lines, and Src is represented as orange diamonds. EPEC: Association with the actin-rich pedestals of noninvasive enteropathogenic *E.coli*. Phagosome: AnxA2 is recruited to actin-rich phagosomes during pigmented retinal epithelial cell phagocytosis of rod outer segments. Macropinosome: AnxA2 is recruited to PI(4,5)P2-rich (purple shading) macropinosomes and is absolutely required for their actin-based rocketing (Comet). Macropinosomes or other early endosomes may mature into APPL2/OCRL1-positive early endosomes. These in turn mature into EEA1-positive endosomes. Endocytic trafficking appears to be involved in formation and reformation of the adherens junction (AJ) and extracellular AnxA2/S110A10 complex, which may be released in exosomes when multivesicular late endosomes (MVBs) fuse with the plasma membrane that may contribute to tight junction stability by cross-linking adjacent membranes. The role of AnxA2 in formation of the apical domain is more speculative but may involve formation of a preformed vacuolar apical compartment (VAC), which then may fuse with the apical surface.

Taken together, we propose that AnxA2 functions at sites of actin association with membranes enriched in cholesterol and/or phosphoinositides. Supporting this proposal, AnxA2 has been shown to have roles in many steps of membrane trafficking and cell polarity. This is reviewed below (also see Figure 2).

## 2. AnxA2 and Membrane Traffic

### 2.1. Formation and Dynamics of Macropinosomes

A number of annexins have been implicated in vesicular trafficking events [53]. A particular function for AnxA2 at the interface of membrane and actin was fully established when it was shown to be required for the rocketing of macropinosomes [54]. These are large vesicles formed by the closure of dynamically ruffling plasma membrane. Although the physiological significance of these objects is debatable, they are likely to be formed by all motile cells (such as macrophages and fibroblasts) and are involved in the process of integrin recycling [55]. Macropinosomes are also formed by professional antigen-presenting cells, which utilise them to sample the extracellular milieu and as a route of entry for a number of viruses. They are produced under a number of different experimental conditions, including the constitutive activation of the small GTPase Rac [56], activation of tyrosine kinase Src [57], or under general enhanced tyrosine and serine phosphorylation (with the use of phosphatase inhibitors) [54]. They also appear under conditions of elevated PI(4,5)P2 production, for example upon phosphatidylinositol 4-5 kinase overexpression or OCRL1 depletion.

 Although AnxA2 is particularly enriched at the junction between the vesicle and the actin, the exact role of AnxA2 in this process is yet to be established. It may act as a barbed-end capping protein, consistent with its enrichment near the vesicle (the site of much of the *de novo* actin polymerization). It may partially restrict the spread and growth of the actin on the comet, to optimize its motility (capping adventitious filaments as they spread from the sides of the actin tail). Alternatively, it may act as a bridge, physically linking the actin to the membrane (a property we have identified for the recombinant protein *in vitro* [42]) or as a bundling protein promoting the condensation of a diffuse actin “halo” into a discrete tail.

### 2.2. Phagocytosis

AnxA2 has also been implicated in the phagocytic process. It accumulates on actin-rich phagosomes produced when outer segments (the effete part of the photoreceptor) are internalized by retinal pigmented epithelial cells, and are lost from the phagosome at the same time as the actin which drives internalization. During phagocytosis, AnxA2 is tyrosine phosphorylated, possibly by Src that is also recruited to the phagocytic cup, suggesting a role for Src-mediated AnxA2 phosphorylation in regulation of the process. Accordingly, depletion of AnxA2 from these cells resulted in slower kinetics of phagocytosis in tissue culture, and AnxA2 knock-out mice show similarly altered kinetics of phagosome degradation [58]. Recently, other annexins including AnxA1 (the most closely related annexin to AnxA2) have also been shown to be involved in phagocytosis and in linking actin to the phagosome [59]. However, a role for the PI(4,5)P2-binding function of AnxA2 in this process has not been ascribed.

### 2.3. Endocytosis

#### 2.3.1. Budding of Clathrin-Coated Vesicles

Annexins have also been shown to be involved in the first step of endocytosis, that is, the formation and pinching off of clathrin-coated vesicles (CCVs). AnxA2 and AnxA6 were first reported on CCVs in the adrenal gland [60]. Association of AnxA2 to these CCVs may be direct through interaction with the *μ*2 subunit of the AP-2 clathrin adaptor complex (identified by two hybrid screening [61]). AP2 is proposed to mediate internalization from the plasma membrane by linking internalizing receptors and cargo to the clathrin coat. *μ*2 binds to the YXX*∅* cytoplasmic domain of transmembrane receptors and AnxA2 has two of these domains in its N-terminus. Furthermore, as the interaction between *μ*2 and YXX*∅* motif of the receptors has been shown to be mediated by PI(4,5)P2 [62], we propose that it may also be the case for AnxA2 given its ability to bind PI(4,5)P2 directly. What importance might AnxA2 have in this event? Actin polymerization has been proposed to transiently occur on invaginating CCVs as they internalize and AnxA2 might help in mediating this transient burst of polymerization. In agreement with this, when AnxA2 is depleted from cells, grape-like clusters of clathrin-coated vesicles are observed [63], suggesting a role for the protein in their budding.

#### 2.3.2. Localization/Positioning of Early and Recycling Endosomes

The first evidence that AnxA2 had a broader role in the endocytic pathway comes from observations by Harder and Gerke and Jost et al., [40, 64] identifying an association of AnxA2 with early endosomes in polarized epithelial cells (Madin-Darby canine kidney cells: MDCK cells). Furthermore, when a chimeric AnxA2/S100A10 fusion protein was introduced into the cells (which caused the aggregation of AnxA2), early endosomes were also aggregated.

AnxA2 association with early endosomes is suggested to be predominantly mediated by its association with cholesterol-rich domains [39]. Indeed, in cells in which cholesterol trafficking is blocked, AnxA2 accumulates on late endosomes and lysosomes (e.g., in Niemann-Pick fibroblasts). This binding was independent of S100A10, indicating that the cholesterol-dependent association of AnxA2 with endosomes does not require formation of the tetrameric complex. However, although the authors of these studies demonstrate continued trafficking of other cargos, gross changes to the trafficking of cholesterol may have unforeseen consequences for the entire endocytic pathway.

 Binding of AnxA2 to endosomes influences their localization. When a chimeric AnxA2/S100A10 fusion protein is introduced into cells (which causes aggregation of AnxA2), early endosomes are also aggregated. Depleting Anx2A also leads to a dramatic change in the general distribution and morphology of early and recycling endosomes that collapse into a perinuclear domain. Furthermore, the tubules of these endosomes become tortuous and bent [63, 64].

Both early and late endosomes have been shown to be actin positive, and AnxA2 has been implicated in regulating this actin [65]. It is tempting to speculate that AnxA2-mediated actin remodeling could have a role to play in maintenance of the architecture and positioning of the recycling endosome. Interestingly, this function is supported by a partial colocalization of AnxA2 with Rab11a (a marker of the recycling endosome) in cells transformed by the oncogene v-Src [66].

#### 2.3.3. Early-to-Late Endosome Maturation

The first indication that AnxA2 was involved in endosomal function came from studies using endosome fusion assays. Indeed, a subtle reduction in endosome fusion mediated by AnxA2-free cytoplasm was noted that was rescued by addition of recombinant protein. Furthermore, HeLa cells depleted of AnxA2 by RNA interference show reduced transfer of a fluid-phase marker of endocytosis and of EGF, to the lysosome, but not internalized LDL [67], suggesting that AnxA2 displays a specific role in trafficking a subset of cargoes.

Conversely, recycling of transferrin receptor is unaffected by AnxA2 loss, suggesting that the protein is involved specifically in maturation of late endosomes [68].

Failure of late endosome maturation could be rescued by a pseudotyrosine-phosphorylated AnxA2 construct but not by a mutant in which the phosphorylation site was mutated [69]. This strongly implicates a role for tyrosine phosphorylation (perhaps by a Src-kinase family member) in regulating AnxA2 function during early-to-late endosome maturation, analogous to the function we propose for AnxA2 in the conversion of APPL endosomes to EEA1 endosomes.

In this respect, Morel et al., [65, 69] recently described dynamic patches of short actin filaments on a subset of both early and late endosomes and crucially, AnxA2 has been shown to be required for formation of these actin patches, as were the actin nucleating proteins Arp2/3 and Spire1. In cells depleted of AnxA2, late endosomes remained intimately associated with the vesiculotubular early endosome. This suggests that AnxA2-mediated actin polymerization may drive a scission event which is in line with a requirement of actin polymerization in the efficient maturation from early to late endosome.

On a speculative note, Anx2 could also have a role in early endosomal conversion. By this, we refer to the existence of a pool of APPL/Rab5-positive but EEA1/PI3P-negative early endosomes that acquire PI3P and EEA1. APPL endosomes are derived from both macropinosomes and a subset of vesicles formed by clathrin-mediated endocytosis and represent a very early stage in the endocytic cycle, lying upstream of the canonical PI(3)P-positive endosomes [70–72]. The distribution of AnxA2 to early endosomes identified by flotation fractionation closely mirrors that of APPL1/2. Furthermore, AnxA2 and APPL1/2 colocalise *in vivo*. Depletion of AnxA2 resulted in reduced formation of APPL-positive endosomes and solubilisation of APPL2 protein. This suggests that AnxA2 plays a key role in the identity and/or formation of APPL-endosomes and warrants further investigation.

Interestingly, APPL proteins physically interact with OCRL1, which is recruited to the CCV after the scission event [73]. This suggests that some function of APPL-endosomes is regulated by dynamic changes in PI(4,5)P2 and/or PI(3,4,5)P3 levels. A negative feedback loop could be envisaged, in which recruitment of AnxA2 to PI(4,5)P2/PI(3,4,5)P3-rich endosomal structures (probably in association with clathrin), is followed by subsequent recruitment of APPL1/2 and OCRL1. The presence of OCRL1 would deplete the early endosome of these phosphoinositides and release AnxA2 and APPL1/2, allowing the endosome to mature towards an EEA1-, PI(3)P-positive fate. This refinement, though extremely speculative, may explain the sometimes-contradictory results reported in the literature and is the subject of ongoing study in our laboratory and others.

## 3. AnxA2 and Epithelial Polarity

The second critical function for AnxA2 that engages both its biochemical properties is exemplified by its role in the establishment of epithelial polarity. In response to signals from the external environment, epithelial cells polarize their membrane domains into distinct apical and basolateral domains, separated by intercellular contacts such as adherens and tight junctions. AnxA2 is able to recruit and potentially activate Rho GTPases as well as key polarity complexes which act in concert to orchestrate the actin cytoskeletal rearrangements required for epithelial polarity. Although some links are yet to be clarified, it is apparent that AnxA2 is a central player in the genesis of epithelial cell polarity.

### 3.1. Annexin A2 Is a Key Orchestrator of Cell-Cell Adhesion through Actin Remodeling

Cell-cell adhesion is strongly regulated by the activities of Rho GTPases and their effects in regulation of the actin cytoskeleton [74, 75]. This requires a balancing act between the different activities of the Rho GTPases, and AnxA2 has been shown to control the localization and activation state of a number of these.

On one hand, AnxA2 recruits Rho to the plasma membrane, for instance in wound closure assays in Caco-2 epithelial cells which facilitates cell motility/migration, a process antagonistic to cell junction formation. Correspondingly, in cells in which AnxA2 is depleted, Rho recruitment to lamellipods is decreased and cells fail to migrate [76].

On the other hand, during the initial stages of cell-cell adhesion, another Rho GTPase, Rac1, becomes localized to nascent junctions, driven by an interaction with E-cadherin [77]. Interestingly, this recruitment of Rac1 is dependent upon AnxA2. The recruitment of AnxA2 to junctions is dependent on PI3K activity, which is consistent with the reported complex between AnxA2 and the regulatory subunit of PI3K [78]. AnxA2 may be recruited to the plasma membrane at sites of nascent junction formation through binding to PI3K directly or by binding to PI(3,4,5)P3, which is in contrast to what is generally assumed to be its preferred phosphoinositide, PI(4,5)P2. It is noteworthy to mention that AnxA2 harbors binding affinity for PI(3,4,5)P3, albeit weaker than that for PI(4,5)P2 [41, 43].

AnxA2 has also been shown to regulate the actin cytoskeleton via its interaction with the scaffold protein, AHNAK (also known as Desmoyokin) [79, 80] which recruited to cholesterol-rich domains of the plasma membrane in an AnxA2-dependent manner. Upon depletion of AnxA2 actin cytoskeleton fails to remodel and epithelial cells cannot mature from a flattened cell state to a characteristic tall columnar state. Depletion of AHNAK alone leads to the same, or at least a very similar, phenotype as AnxA2 depletion.

### 3.2. AnxA2 Is Required for Adherens Junction Formation

A second critical role for AnxA2 in cell polarity is in the formation of E-cadherin-based adhesions downstream of Nectin-1. In a so-called “calcium switch”-experiment in which cell-cell contacts are disassembled and then can be observed as they are rebuilt over time, rerecruitment of E-cadherin to junctions fails in cells depleted of AnxA2. Despite the lack of E-cadherin targeting, the tight junction still is formed in these cells [81]. This clearly indicates that AnxA2 has a key role in the establishment of E-cadherin-based adherens junctions but not tight junctions. These data place AnxA2 at the heart of nascent E-cadherin-based adhesions, which are critical to the underlying actin cytoskeletal network of epithelial cells.

The role of AnxA2 is not limited to epithelial, but also endothelial cells. AnxA2 has been shown to play a key role in the structure of endothelial adherens junctions and associates with VE-cadherin [82, 83]. Depletion of AnxA2 results in the loss of VE-cadherin from junctions leading to subsequent adherens junction instability. Endothelial morphogenesis is severely disrupted in these cells, due to inhibition of an AKT signaling pathway downstream of the adherens junction [82]. In addition, VEGF treatment destabilizes the interaction between AnxA2 and VE-cad and results in subsequent loss of junctional integrity. This may seem inconsistent with the apparently normal vasculature of the AnxA2 knock-out mouse, suggesting a redundancy in vascular development that ensures the proper formation of junctions even in the absence of AnxA2. The AnxA2 knock-out mouse does, however, demonstrate defects in neoangiogenesis [18], a related process that provides tumours with the necessary blood flow for growth. Indeed, AnxA2 KO mice are incapable of supporting the growth of solid tumours in an implantation assay, consistent with a role for AnxA2 in tumour progression.

### 3.3. 3D Cysts Reveal a Key Contribution of AnxA2 in the Establishment of Epithelial Organs

As described above, AnxA2 functions as an initiator of actin remodeling events and does so through the integration of spatiotemporal modulations of phosphoinositides and the recruitment and/or activation of Rho GTPases. In addition, use of 3D MDCK cysts reveal key findings that depletion of AnxA2 or overexpression of dominant negative AnxA2 prevents the formation of the central lumen [84]. AnxA2 is thought to influence this process on two critical sites: at Rab11a-positive recycling endosomes and at the plasma membrane.

#### 3.3.1. A Role for AnxA2 at Rab11a Endosomes in Lumen Formation

Rab11a recycling endosomes are now recognized as a key intermediate in the trafficking of junctional proteins, such as E-cadherin, to the plasma membrane [85–87]. AnxA2 transiently localizes to Rab11a endosomes that in epithelial cells contain the apical marker GP135/podocalyxin, prior to lumen formation [87, 88]. Overexpression of a dominant negative form of AnxA2 leads to defects in the development of the lumen that coincides with the accumulation of GP135 in Rab11a endosomes, suggesting that AnxA2 plays a key role in the delivery of this marker from a “recycling compartment” to the plasma membrane. It appears that AnxA2 has a role in GP135 recycling in spite of having no obvious effect on transferrin recycling (see Section 2.3). This may reflect specific functions related to cell types and cargo.

#### 3.3.2. Annexin A2 and Polarized Plasma Membrane Domains

In addition to recycling of apical markers, AnxA2 plays a key role at polarized plasma membrane domains. During the formation of 3D MDCK cysts, prior to lumenogenesis, PI(3,4,5)P3 is enriched at the plasma membrane, relative to PI(4,5)P2 [84, 89]. At that stage, AnxA2 localizes to the plasma membrane, perhaps through a weak interaction with PI(3,4,5)P3 [44].

Vital to the formation of a lumen is the activity of the inositol lipid 3′-phosphatase PTEN, which is recruited to the presumptive apical surface where it generates PI(4,5)P2 which AnxA2 binds to with a higher affinity. Upon generation of PI(4,5)P2 at apical membranes by PTEN, AnxA2 shifts its localization to sites at which its preferred substrate is concentrated [84].

AnxA2 binding to PI(4,5)P2 at the apical surface initiates a cascade of events vital to epithelial morphogenesis. For instance, AnxA2 is proposed to recruit another Rho-subfamily member, Cdc42, and the key polarity proteins aPKC and Par6 to the nascent apical surface [84, 88].

 Although it seems most likely that AnxA2 functions to scaffold active Cdc42 and nucleate actin remodeling, it is also highly possible that the formation of the apical surface requires the fusion of small vesicles enriched with AnxA2. Interestingly, Cdc42 is required for TGN exit of basolateral proteins [90] and AnxA2 has been detected in TGN-derived apically targeted vesicles in MDCK cells. In this context, downregulation of AnxA2 in MDCK cells specifically interferes with the routing of vesicles to the apical plasma membrane [91]. This has also been demonstrated in Caco-2 cells, in which depletion of AnxA2 leads to a strong polarization defect, characterized by a morphological transition to flat, undifferentiated epithelial cells with sparse and short microvilli and reduced levels of brush border proteins [92]. Interestingly, myristoylated annexin XIIIB has also been shown to be essential for apical delivery of such vesicles and revealed to be vital to 3D cyst formation [93–95]. This may explain why the phenotype of AnxA2 RNAi in cysts is not as penetrant as overexpression of dominant negative forms of it or as dramatic as depletions of other molecular components required for cyst formation such as Cdc42, aPKC, the exocyst, and PTEN.

### 3.4. A Role for Extracellular AnxA2 in Cell-Cell Adhesion

Not only AnxA2 is expressed at particularly high levels in epithelia and endothelia intracellularly, but it also appears as an extracellular, cell-surface-associated pool in complex with S100A10. How the tetrameric complex exits cells to appear on the outer leaflet has also been a subject of much discussion. As a protein lacking a signal peptide, AnxA2 is one of a growing list of unconventionally secreted proteins [96–98], which could occur by one of three ways. The first is by incorporation into intraluminal vesicles of multivesicular endosomes and subsequent release as exosomes [99]. This process is promoted by interaction with S100A10 and by phosphorylation at Tyr23 [100]. The second emanates from a study of the short-lived cells of the enterocyte brush border, which are covered in AnxA2 on their luminal, microvillus-decorated surface [101]. In these cells, AnxA2 nonconventional secretion is proposed to involve an intimate association with SNARE proteins at sites of endosome/plasma membrane fusion. This suggests that SNARE-mediated fusion of the membranes forces the annexin complex across the membrane and out of the cell. The third would be similar to what has been shown for another unconventionally secreted protein, FGF-2, whereby it exits cells by forming a transmembrane channel or pore [98]. Interestingly, this pore formation is strongly regulated by the presence of PI(4,5)P2 at the plasma membrane, a feature that fits the biochemical properties of AnxA2.

Whichever the means of cell egress, extracellular AnxA2 has been implicated in in membrane bridging [102, 103] and tight junction formation [104] and may represent an additional mode of regulating epithelial morphogenesis to that we described earlier in Section 3.

Tetrameric AnxA2 complex colocalises with tight junction markers along the lateral plasma membrane and a pool of this protein was shown to be extracellular. Addition of an inhibitory peptide (aimed at interrupting the AnxA2/S100A10 complex formation) across a calcium switch experiment blocked tight junction reformation, consistent with the idea that extracellular AnxA2 plays an important role in remodeling of tight junctions [104].

Phosphorylation of AnxA2 on Tyr23 leads to its increased recruitment to the plasma membrane and subsequently to sites of actin remodeling [105]. Given that it also promotes AnxA2 secretion, it would be of interest to analyze the relative contribution of this secreted AnxA2 on MDCK cell scattering, in comparison to the known intracellular functions of AnxA2.

## 4. Discussion

The principal biochemical properties of AnxA2 are calcium-dependent association with phospholipids and actin. These properties have now been refined both *in vitro* and *in vivo* at physiologically significant calcium concentrations to an affinity for PI(4,5)P2-, PI(3,4,5)P3- and/or cholesterol-containing membranes and the ability to cap the fast-growing ends of actin filaments. These are subtle but important refinements because they change the role of the protein from being that of a structural “contaminant” of membrane and cytoskeletal preparations to being critical in establishing a specific subset of membrane-actin interfaces. It may be the case that the annexin itself is sufficient to form this structure but it is likely that *in vivo* a number of annexin/actin/PI(4,5)P2-associated proteins ultimately bulwark the membrane/actin interface.

 In this paper, we have outlined the predominant role of AnxA2 in events that occur in the vicinity of the plasma membrane including clathrin-mediated budding, formation of macropinosomes and phagosomes, conversion of early endosomes and maturation of early-to-late endosomes (depicted in Figure 2). AnxA2 is also important for secretory events, such as granule secretion. Furthermore, the formation of the apical domain and cell-cell junctions in polarized cells is also highly dependent upon AnxA2.

AnxA2 may mediate these events (almost all of which are mediated by PI(4,5)P2 and calcium) either as a result of its inherent biological activity or indirectly through its *interactome* (e.g., the recruitment of small Rho GTPases or potential interactions with actin/lipid metabolizing proteins). The potential role for AnxA2 at recycling endosome events and during endosome-maturation is less well established and may be a cholesterol-mediated function or occur under specific conditions, such as when the protein is hyper-phosphorylated, for example during Src-mediated transformation.

Almost all these processes occur concomitantly with significant actin remodeling. This may be necessary to drive vesicle motility or to ease endosome movement through dense cortical actin filaments. However, the importance of short bursts of actin polymerization during vesicle fusion and fission is controversial and is less well understood with regard to the maintenance of endosomal structure.

 In the formation and motility of actin rockets and macropinosomes and in 3D epithelial polarity, both lipid and actin binding properties of AnxA2 have been implicated. In other events linked to AnxA2, only one of its biochemical properties has so far been involved. For instance, in our proposed model of early endosome transition, processes of CCV formation, and granule secretion, there is clearly a role for PI(4,5)P2 dynamics; but a role for actin is less well established. Conversely, the maturation of early-to-late endosomes requires AnxA2-mediated actin polymerization, but a role for PI(4,5)P2 regulation of AnxA2 in this process is still to be demonstrated. There is clearly room for further investigations of the link between the two biochemical properties of AnxA2 in these processes.

 AnxA2 is highly abundant in those cells in which it is expressed; but it is not ubiquitous. For this reason, it cannot be critical in all cell types for all the processes we have described in this paper. This may be partly explained by the potential redundancy between annexin family members that due to space limitation, we have not been able to discuss. The Annexin family is highly conserved and many of the other members, in spite of having unique sequences (e.g., in their N-termini) have shared biochemical activities with AnxA2. Individual lipid/calcium/actin-binding affinities and specificities may vary, but the potential for overlap is clear. Indeed, in many cases, they have been implicated in related processes and may form oligomeric complexes; though as yet overexpression of one family member has not been shown to compensate for loss of another. This may explain the viability and fertility of the AnxA2 knockout mouse. Notwithstanding homeostatic alterations in annexin or other gene expression which may compensate for genetic lesion, the mouse does seem surprisingly healthy given the number of processes in which AnxA2 has been implicated. It appears that the major defects in the mouse are not in normal development; but rather in subsequent plastic changes: the mouse seems to be incapable of sustaining neovascularisation, for example, which may make AnxA2 a promising target for drug intervention in cancer treatment. Inherent redundancies in endocytosis, phagocytosis, and junction formation may mean that AnxA2 phenotypes are not as clear in the developing animal as they seem to be in culture. The AnxA2 mouse is also effectively an S100A10 knockout animal as AnxA2 serves to prevent S100A10 degradation in the cytoplasm. Overexpression of AnxA2 (as is seen in some cancers) or nonstoichiometric changes in the relative concentrations of AnxA2 and S100A10 may have more severe phenotypes than loss of both.

There are still a number of important gaps in the AnxA2 story, such as a lack of AnxA2 crystal structure in complex with either actin or PI(4,5)P2 or how phosphorylation of the protein (e.g., by Src) or PI(4,5)P2 binding precisely affects its actin remodeling activity and membrane dynamics. We can hope that further research will throw more light on the role of this vital cellular component.

As a final note, alterations in AnxA2 expression or activity have been demonstrated in many different cancers, particularly those of the prostate, the breast, the liver, the lung (there are too many references to cite meaningfully here), and in the formation of infective HIV particles [29, 30]. Exactly what the protein is doing in these contexts is a subject of much speculation; but given the importance of endocytic trafficking and cell polarization in these diseases, it is likely that some of the factors described above will contribute to this role.

## References

[1] Gerke V, Moss SE (2002). Annexins: from structure to function. *Physiological Reviews*.

[2] Moss SE, Morgan RO (2004). The annexins. *Genome Biology*.

[3] Gerke V, Creutz CE, Moss SE (2005). Annexins: linking Ca^2+^ signalling to membrane dynamics. *Nature Reviews Molecular Cell Biology*.

[4] Rescher U, Gerke V (2004). Annexins—unique membrane binding proteins with diverse functions. *Journal of Cell Science*.

[5] Mortimer JC, Laohavisit A, Macpherson N (2008). Annexins: multifunctional components of growth and adaptation. *The Journal of Experimental Botany*.

[6] Laohavisit A, Davies JM (2011). Annexins. *New Phytologist*.

[7] Konopka-Postupolska D, Clark G, Hofmann A (2011). Structure, function and membrane interactions of plant annexins: an update. *Plant Science*.

[8] Hawkins TE, Merrifield CJ, Moss SE (2000). Calcium signaling and annexins. *Cell Biochemistry and Biophysics*.

[9] Monastyrskaya K, Babiychuk EB, Hostettler A, Rescher U, Draeger A (2007). Annexins as intracellular calcium sensors. *Cell Calcium*.

[10] van de Graaf SFJ, Hoenderop JGJ, Bindels RJM (2006). Regulation of TRPV5 and TRPV6 by associated proteins. *American Journal of Physiology*.

[11] Monastyrskaya K, Tschumi F, Babiychuk EB, Stroka D, Draeger A (2008). Annexins sense changes in intracellular pH during hypoxia. *Biochemical Journal*.

[12] Hayes MJ, Rescher U, Gerke V, Moss SE (2004). Annexin-actin interactions. *Traffic*.

[13] Konopka-Postupolska D (2007). Annexins: putative linkers in dynamic membrane-cytoskeleton interactions in plant cells. *Protoplasma*.

[14] Grewal T, Enrich C (2009). Annexins—modulators of EGF receptor signalling and trafficking. *Cellular Signalling*.

[15] John CD, Christian HC, Morris JF, Flower RJ, Solito E, Buckingham JC (2004). Annexin 1 and the regulation of endocrine function. *Trends in Endocrinology and Metabolism*.

[16] Buckingham JC, John CD, Solito E (2006). Annexin 1, glucocorticoids, and the neuroendocrine-immune interface. *Annals of the New York Academy of Sciences*.

[17] Reutelingsperger CPM (2001). Annexins: key regulators of haemostasis, thrombosis, and apoptosis. *Thrombosis and Haemostasis*.

[18] Ling Q, Jacovina AT, Deora A (2004). Annexin II regulates fibrin homeostasis and neoangiogenesis in vivo. *The Journal of Clinical Investigation*.

[19] Kassam G, Choi KS, Ghuman J (1998). The role of annexin II tetramer in the activation of plasminogen. *The Journal of Biological Chemistry*.

[20] Choi KS, Ghuman J, Kassam G, Kang HM, Fitzpatrick SL, Waisman DM (1998). Annexin II tetramer inhibits plasmin-dependent fibrinolysis. *Biochemistry*.

[21] Rand JH (2000). The annexinopathies: a new category of diseases. *Biochimica et Biophysica Acta*.

[22] Fatimathas L, Moss SE (2010). Annexins as disease modifiers. *Histology and Histopathology*.

[23] Hayes MJ, Moss SE (2004). Annexins and disease. *Biochemical and Biophysical Research Communications*.

[24] Hayes MJ, Longbottom RE, Evans MA, Moss SE (2007). Annexinopathies. *Subcellular Biochemistry*.

[25] Rand JH, Wu XX (2004). Antibody-mediated disruption of the annexin-V antithrombotic shield: a new mechanism for thrombosis in the antiphospholipid syndrome. *Thrombosis Research*.

[26] Balcerzak M, Hamade E, Zhang L (2003). The roles of annexins and alkaline phosphatase in mineralization process. *Acta Biochimica Polonica*.

[27] Kirsch T (2005). Annexins—their role in cartilage mineralization. *Frontiers in Bioscience*.

[28] Mussunoor S, Murray GI (2008). The role of annexins in tumour development and progression. *The Journal of Pathology*.

[29] Harrist AV, Ryzhova EV, Harvey T, González-Scarano F (2009). Anx2 interacts with HIV-1 Gag at phosphatidylinositol (4,5) bisphosphate-containing lipid rafts and increases viral production in 293T cells. *PLoS One*.

[30] Rai T, Mosoian A, Resh MD (2010). Annexin 2 is not required for human immunodeficiency virus type 1 particle production but plays a cell type-dependent role in regulating infectivity. *Journal of Virology*.

[31] Glenney JR (1985). Phosphorylation of p36 in vitro with pp60src. Regulation by Ca^2+^ and phospholipid. *FEBS Letters*.

[32] Glenney JR, Tack BF (1985). Amino-terminal sequence of p36 and associated p10: identification of the site of tyrosine phosphorylation and homology with S-100. *Proceedings of the National Academy of Sciences of the United States of America*.

[33] Saris CJ, Tack BF, Kristensen T, Glenney JR, Hunter T (1986). The cDNA sequence for the protein-tyrosine kinase substrate p36 (calpactin I heavy chain) reveals a multidomain protein with internal repeats. *Cell*.

[34] Powell MA, Glenney JR (1987). Regulation of calpactin I phospholipid binding by calpactin I light-chain binding and phosphorylation by p60(v-src). *Biochemical Journal*.

[35] Zokas L, Glenney JR (1987). The calpactin light chain is tightly linked to the cytoskeletal form of calpactin I: studies using monoclonal antibodies to calpactin subunits. *The Journal of Cell Biology*.

[36] Osborn M, Johnsson N, Wehland J, Weber K (1988). The submembranous location of p11 and its interaction with the p36 substrate of pp60 src kinase in situ. *Experimental Cell Research*.

[37] Semich R, Gerke V, Robenek H, Weber K (1989). The p36 substrate of pp60(src) kinase is located at the cytoplasmic surface of the plasma membrane of fibroblasts; an immunoelectron microscopic analysis. *European Journal of Cell Biology*.

[38] Oliferenko S, Paiha K, Harder T (1999). Analysis of CD44-containing lipid rafts: recruitment of annexin II and stabilization by the actin cytoskeleton. *The Journal of Cell Biology*.

[39] Harder T, Kellner R, Parton RG, Gruenberg J (1997). Specific release of membrane-bound annexin II and cortical cytoskeletal elements by sequestration of membrane cholesterol. *Molecular Biology of the Cell*.

[40] Jost M, Zeuschner D, Seemann J, Weber K, Gerke V (1997). Identification and characterization of a novel type of annexin-membrane interaction: Ca^2+^ is not required for the association of annexin II with early endosomes. *Journal of Cell Science*.

[41] Hayes MJ, Merrifield CJ, Shao D (2004). AnxA2 binding to phosphatidylinositol 4,5-bisphosphate on endocytic vesicles is regulated by the stress response pathway. *The Journal of Biological Chemistry*.

[42] Hayes MJ, Shao D-M, Grieve A, Levine T, Bailly M, Moss SE (2009). Annexin A2 at the interface between F-actin and membranes enriched in phosphatidylinositol 4,5,-bisphosphate. *Biochimica et Biophysica Acta*.

[43] Rescher U, Ruhe D, Ludwig C, Zobiack N, Gerke V (2004). Annexin 2 is a phosphatidylinositol (4,5)-bisphosphate binding protein recruited to actin assembly sites at cellular membranes. *Journal of Cell Science*.

[44] Gokhale NA, Abraham A, Digman MA, Gratton E, Cho W (2005). Phosphoinositide specificity of and mechanism of lipid domain formation by annexin A2-p11 heterotetramer. *The Journal of Biological Chemistry*.

[45] Allen PG (2003). Actin filament uncapping localizes to ruffling lamellae and rocketing vesicles. *Nature Cell Biology*.

[46] Lowe M (2005). Structure and function of the Lowe syndrome protein OCRL1. *Traffic*.

[47] Gerke V, Weber K (1984). Identity of p36K phosphorylated upon Rous sarcoma virus transformation with a protein purified from brush borders; calcium-dependent binding to non-erythroid spectrin and F-actin. *EMBO Journal*.

[48] Glenney JR (1986). Two related but distinct forms of the Mr 36,000 tyrosine kinase substrate (calpactin) that interact with phospholipid and actin in a Ca^2+^-dependent manner. *Proceedings of the National Academy of Sciences of the United States of America*.

[49] Ikebuchi NW, Waisman DM (1990). Calcium-dependent regulation of actin filament bundling by lipocortin-85. *The Journal of Biological Chemistry*.

[50] Jones PG, Moore GJ, Waisman DM (1992). A nonapeptide to the putative F-actin binding site of annexin-II tetramer inhibits its calcium-dependent activation of actin filament bundling. *The Journal of Biological Chemistry*.

[51] Hubaishy I, Jones PG, Bjorge J (1995). Modulation of annexin II tetramer by tyrosine phosphorylation. *Biochemistry*.

[52] Hayes MJ, Shao D, Bailly M, Moss SE (2006). Regulation of actin dynamics by annexin 2. *EMBO Journal*.

[53] Futter CE, White IJ (2007). Annexins and endocytosis. *Traffic*.

[54] Merrifield CJ, Rescher U, Almers W (2001). Annexin 2 has an essential role in actin-based macropinocytic rocketing. *Current Biology*.

[55] Gu Z, Noss EH, Hsu VW, Brenner MB (2011). Integrins traffic rapidly via circular dorsal ruffles and macropinocytosis during stimulated cell migration. *The Journal of Cell Biology*.

[56] Fumoto S, Nishi J, Ishii H (2009). Rac-mediated macropinocytosis is a critical route for naked plasmid DNA transfer in mice. *Molecular Pharmaceutics*.

[57] Veithen A, Cupers P, Baudhuin P, Courtoy PJ (1996). v-Src induces constitutive macropinocytosis in rat fibroblasts. *Journal of Cell Science*.

[58] Law AL, Ling Q, Hajjar KA (2009). Annexin A2 regulates phagocytosis of photoreceptor outer segments in the mouse retina. *Molecular Biology of the Cell*.

[59] Patel DM, Ahmad SF, Weiss DG, Gerke V, Kuznetsov SA (2011). Annexin A1 is a new functional linker between actin filaments and phagosomes during phagocytosis. *Journal of Cell Science*.

[60] Turpin E, Russo-Marie F, Dubois T, de Paillerets C, Alfsen A, Bomsel M (1998). In adrenocortical tissue, annexins II and VI are attached to clathrin coated vesicles in a calcium-independent manner. *Biochimica et Biophysica Acta*.

[61] Creutz CE, Snyder SL (2005). Interactions of annexins with the mu subunits of the clathrin assembly proteins. *Biochemistry*.

[62] Höning S, Ricotta D, Krauss M (2005). Phosphatidylinositol-(4,5)-bisphosphate regulates sorting signal recognition by the clathrin-associated adaptor complex AP2. *Molecular Cell*.

[63] Zobiack N, Rescher U, Ludwig C, Zeuschner D, Gerke V (2003). The AnxA2/S100A10 complex controls the distribution of transferrin receptor-containing recycling endosomes. *Molecular Biology of the Cell*.

[64] Harder T, Gerke V (1993). The subcellular distribution of early endosomes is affected by the annexin II2p11(2) complex. *The Journal of Cell Biology*.

[65] Morel E, Parton RG, Gruenberg J (2009). Annexin A2-dependent polymerization of actin mediates endosome biogenesis. *Developmental Cell*.

[66] Hayes MJ, Moss SE (2009). Annexin 2 has a dual role as regulator and effector of v-Src in cell transformation. *The Journal of Biological Chemistry*.

[67] Mayran N, Parton RG, Gruenberg J (2003). Annexin II regulates multivesicular endosome biogenesis in the degradation pathway of animal cells. *EMBO Journal*.

[68] Zobiack N, Rescher U, Ludwig C, Zeuschner D, Gerke V (2003). The annexin 2/S100A10 complex controls the distribution of transferrin receptor-containing recycling endosomes. *Molecular Biology of the Cell*.

[69] Morel E, Gruenberg J (2009). Annexin A2 Binding to endosomes and functions in endosomal transport are regulated by tyrosine 23 phosphorylation. *The Journal of Biological Chemistry*.

[70] Bucci C, Parton RG, Mather IH (1992). The small GTPase rab5 functions as a regulatory factor in the early endocytic pathway. *Cell*.

[71] Zoncu R, Perera RM, Balkin DM, Pirruccello M, Toomre D, De Camilli P (2009). A phosphoinositide switch controls the maturation and signaling properties of APPL endosomes. *Cell*.

[72] Urbanska A, Sadowski L, Kalaidzidis Y, Miaczynska M (2011). Biochemical characterization of APPL endosomes: the role of AnxA2 in APPL membrane recruitment. *Traffic*.

[73] Erdmann KS, Mao Y, McCrea HJ (2007). . A role of the Lowe syndrome protein OCRL in early steps of the endocytic pathway. *Developmental Cell*.

[74] Tapon N, Hall A (1997). Rho, Rac and Cdc42 GTPases regulate the organization of the actin cytoskeleton. *Current Opinion in Cell Biology*.

[75] Hall A (1998). Rho GTpases and the actin cytoskeleton. *Science*.

[76] Babbin BA, Parkos CA, Mandell KJ (2007). Annexin 2 regulates intestinal epithelial cell spreading and wound closure through rho-related signaling. *American Journal of Pathology*.

[77] Hansen MD, Ehrlich JS, Nelson WJ (2002). Molecular mechanism for orienting membrane and actin dynamics to nascent cell-cell contacts in epithelial cells. *The Journal of Biological Chemistry*.

[78] Barwe SP, Anilkumar G, Moon SY (2005). Novel role for Na,K-ATPase in phosphatidylinositol 3-kinase signaling and suppression of cell motility. *Molecular Biology of the Cell*.

[79] Benaud C, Gentil BJ, Assard N (2004). AHNAK interaction with the annexin 2/S100A10 complex regulates cell membrane cytoarchitecture. *The Journal of Cell Biology*.

[80] De Seranno S, Benaud C, Assard N (2006). Identification of an AHNAK binding motif specific for the Annexin2/S100A10 tetramer. *The Journal of Biological Chemistry*.

[81] Yamada A, Irie K, Hirota T, Ooshio T, Fukuhara A, Takai Y (2005). Involvement of the annexin II-S100A10 complex in the formation of E-cadherin-based adherens junctions in madin-darby canine kidney cells. *The Journal of Biological Chemistry*.

[82] Su SC, Maxwell SA, Bayless KJ (2010). Annexin 2 regulates endothelial morphogenesis by controlling AKT activation and junctional integrity. *The Journal of Biological Chemistry*.

[83] Heyraud S, Jaquinod M, Durmort C (2008). Contribution of annexin 2 to the architecture of mature endothelial adherens junctions. *Molecular and Cellular Biology*.

[84] Martin-Belmonte F, Gassama A, Datta A (2007). PTEN-mediated apical segregation of phosphoinositides controls epithelial morphogenesis through Cdc42. *Cell*.

[85] Ivanov AI, Nusrat A, Parkos CA (2004). Endocytosis of epithelial apical junctional proteins by a clathrin-mediated pathway into a unique storage compartment. *Molecular Biology of the Cell*.

[86] Desclozeaux M, Venturato J, Wylie FG (2008). Active Rab11 and functional recycling endosome are required for E-cadherin trafficking and lumen formation during epithelial morphogenesis. *American Journal of Physiology*.

[87] Lock JG, Stow JL (2005). Rab11 in recycling endosomes regulates the sorting and basolateral transport of E-cadherin. *Molecular Biology of the Cell*.

[88] Bryant DM, Datta A, Rodríguez-Fraticelli AE, PeräCurrency Signnen J, Martín-Belmonte F, Mostov KE (2010). A molecular network for de novo generation of the apical surface and lumen. *Nature Cell Biology*.

[89] Martin-Belmonte F, Mostov K (2007). Phosphoinositides control membrane polarity in epithelial development. *Cell Cycle*.

[90] Malacombe M, Ceridono M, Calco V (2006). Intersectin-1L nucleotide exchange factor regulates secretory granule exocytosis by activating Cdc42. *EMBO Journal*.

[91] Jacob R, Heine M, Eikemeyer J (2004). Annexin II is required for apical transport in polarized epithelial cells. *The Journal of Biological Chemistry*.

[92] Hein Z, Schmidt S, Zimmer KP, Naim HY (2011). The dual role of annexin II in targeting of brush border proteins and in intestinal cell polarity. *Differentiation*.

[93] Lafont F, Lecat S, Verkade P, Simons K (1998). Annexin XIIIb associates with lipid microdomains to function in apical delivery. *The Journal of Cell Biology*.

[94] Fiedler K, Lafont F, Parton RG, Simons K (1995). Annexin XIIIb: a novel epithelial specific annexin is implicated in vesicular traffic to the apical plasma membrane.. *The Journal of Cell Biology*.

[95] Torkko JM, Manninen A, Schuck S, Simons K (2008). Depletion of apical transport proteins perturbs epithelial cyst formation and ciliogenesis. *Journal of Cell Science*.

[96] Giuliani F, Grieve A, Rabouille C (2011). Unconventional secretion: a stress on GRASP. *Current Opinion in Cell Biology*.

[97] Nickel W (2011). The unconventional secretory machinery of fibroblast growth factor 2. *Traffic*.

[98] Temmerman K, Ebert AD, Müller HM, Sinning I, Tews I, Nickel W (2008). A direct role for phosphatidylinositol-4,5-bisphosphate in unconventional secretion of fibroblast growth factor 2. *Traffic*.

[99] Valapala M, Vishwanatha JK (2011). Lipid raft endocytosis and exosomal transport facilitate extracellular trafficking of annexin A2. *The Journal of Biological Chemistry*.

[100] Deora AB, Kreitzer G, Jacovina AT, Hajjar KA (2004). An annexin 2 phosphorylation switch mediates p11-dependent translocation of annexin 2 to the cell surface. *The Journal of Biological Chemistry*.

[101] Danielsen EM, van Deurs B, Hansen GH (2003). ‘Nonclassical’ secretion of annexin A2 to the lumenal side of the enterocyte brush border membrane. *Biochemistry*.

[102] Ayala-Sanmartin J, Zibouche M, Illien F, Vincent M, Gallay J (2008). Insight into the location and dynamics of the annexin A2 N-terminal domain during Ca^2+^-induced membrane bridging. *Biochimica et Biophysica Acta*.

[103] Zibouche M, Vincent M, Illien F, Gallay J, Ayala-Sanmartin J (2008). The N-terminal domain of annexin 2 serves as a secondary binding site during membrane bridging. *The Journal of Biological Chemistry*.

[104] Lee DBN, Jamgotchian N, Allen SG, Kan FWK, Hale IL (2004). Annexin A2 heterotetramer: role in tight junction assembly. *American Journal of Physiology*.

[105] Rescher U, Ludwig C, Konietzko V, Kharitonenkov A, Gerke V (2008). Tyrosine phosphorylation of annexin A2 regulates Rho-mediated actin rearrangement and cell adhesion. *Journal of Cell Science*.

